# Molecular Targets and Trait Innovation in CRISPR-Edited Vegetable Crops: An Up-to-Date Review (2020–2026)

**DOI:** 10.3390/plants15182885

**Published:** 2026-09-21

**Authors:** Eleftheria Deligiannidou, Ioannis Ganopoulos, Vasileios Papasotiropoulos

**Affiliations:** 1 Laboratory of Plant Breeding and Biometry, Department of Crop Science, Agricultural University of Athens, 11855 Athens, Attica, Greece; rikadel22@gmail.com; 2 Institute of Plant Breeding and Genetic Resources, Hellenic Agricultural Organization-ELGO DIMITRA, Thermi-Thermi Road, 57001 Thermi, Thessaloniki, Greece

**Keywords:** CRISPR/Cas, genome editing, vegetable crops, new genomic techniques, biotic stress, abiotic stress, tomato, potato, plant biotechnology

## Abstract

Vegetable crops are of fundamental nutritional and economic importance worldwide, yet their improvement through conventional breeding remains time consuming and constrained by genomic complexity. For that reason, over the period 2020–2026, CRISPR/Cas-mediated genome editing, a new genomic technique (NGT), has emerged as the leading tool for precise, rapid and targeted modification of vegetable crop genomes. In this review, we summarize selected peer-reviewed studies on CRISPR/Cas applications across a broad range of vegetable species. Tomato (*Solanum lycopersicum*) and potato (*Solanum tuberosum*) are the most edited species with the CRISPR/Cas system, with tomato being at the forefront, while other crops such as cucumber, watermelon, *Brassica* species, pepper, eggplant and lettuce, follow. We studied eight trait categories that have been improved, namely biotic stress resistance, abiotic stress resistance, yield and growth regulation, food/feed quality modification, color/flavor modification, storage conditions, herbicide resistance and industrial utilization. Out of these, biotic stress resistance is the most actively targeted category across both tomato and potato, with viral, fungal, bacterial and oomycete pathogens being addressed through the editing of host susceptibility genes. Some advances include editing *SlDMR6-1* and *DMR6* orthologs to confer broad-spectrum disease resistance, multiplex editing of the *MLO* gene family to achieve powdery mildew resistance, the utilization of the CRISPR/Cas13 system to confer resistance in RNA viruses in potato, and the engineering of drought-tolerant and quality-improved varieties across multiple species. Finally, the regulatory landscape for genome-edited products in the European Union is currently evolving with efforts to distinguish them from genome-modified products and drive further growth for the field.

## 1. Introduction

Vegetable crops are widely produced for human consumption because they are a valuable and nutritious source of vitamins, minerals, antioxidant compounds, fiber and carbohydrates [1]. At the same time, global vegetable production faces multiple challenges, caused by biotic stresses, such as fungal, bacterial and viral pathogens, and by abiotic stresses, including drought, heat and soil salinity [2,3]. Climate change has particularly affected the yield, quality, and productivity of vegetable crops, either by increasing the risk of sudden crop loss or by causing a continuous decline in yields due to fluctuations in environmental conditions [4]. Crop diseases alone are estimated to cause annual agricultural yield losses of 20–40% [2,3], while climate-related uncertainties can impose substantial economic hardships on small-scale and subsistence farmers [5].

New genomic techniques (NGTs), also known as precision breeding techniques, can be applied in various ways to achieve a range of results and products. They have transformed the landscape of crop improvement by producing organisms that could have arisen through conventional breeding, or organisms that have undergone more complex editing, encompassing methods such as targeted mutagenesis, cisgenesis, and intragenesis [6]. These techniques edit the genetic material of plants, animals, or microorganisms by adding, removing, or rearranging DNA sections, with the goal to create new traits or modify existing ones. Among NGTs, the Clustered Regularly Interspaced Short Palindromic Repeats/Cas (CRISPR/Cas) system has rapidly become the dominant tool because of its simplicity, versatility and precision [7,8]. In contrast to earlier editing platforms such as Zinc Finger Nucleases (ZFNs) and Transcription Activator-Like Effector Nucleases (TALENs), CRISPR/Cas achieves target modification using a single guide RNA (sgRNA), free from the need for laborious protein engineering [9]. More specifically, CRISPR/Cas9 technology is derived from the CRISPR-Cas adaptive bacterial immune system. The Cas9 endonuclease uses an RNA duplex, the trans-activating CRISPR RNA:CRISPR RNA (tracrRNA:crRNA), to locate complementary target sequences and introduce site-specific double-strand breaks in DNA. Target recognition requires both base pairing between the crRNA and the target sequence and the presence of the protospacer adjacent motif (PAM), a short sequence adjacent to the targeted sequence in the DNA. The natural duplex tracrRNA:crRNA was subsequently engineered into a sgRNA, which both determines the DNA target site and binds to Cas9. As a result, by altering the guide sequence of the sgRNA, Cas9 can be redirected to any genomic locus of interest, provided a PAM sequence is present in the adjacent DNA [10].

Unlike traditional breeding, where variability mainly occurs from spontaneous or chemically induced mutations, genome editing enables precise, targeted modifications at specific genomic loci in any crop species within a single event [8]. Conventional breeding requires 5–6 generations and a minimum of 7–10 years to transfer a trait within a species. Through biotechnological approaches, this can be reduced to approximately 5–6 years, and in vegetable crops the timeline is even shorter, requiring around two generations [11]. Furthermore, the long domestication process of modern crops has led to genetic bottlenecks, which complicates the further improvement of elite varieties through traditional means. CRISPR/Cas accelerates crop improvement by allowing breeders to directly introduce beneficial genetic variants, including those found in wild relatives, into elite cultivated varieties, without the time and genetic drag associated with conventional crossing and selection [12,13]. This is possible because CRISPR/Cas is a system with diverse Cas variants, comprising types II (Cas9, etc.) and V (Cas12a, Cas12b, Cas12e, Cas14, etc.) for DNA editing and type VI for RNA editing (Cas13, etc.) [14], that can be applied in multiple ways to perform a range of editing strategies, including knockout mutagenesis, multiplex editing, base editing, prime editing, transposase system, de novo domestication, and fine-tuning of gene expression through CRISPRa/CRISPRi systems (Table 1).

Among these, knockout mutagenesis relies on a nuclease cutting a target gene and triggering error-prone repair that disrupts its function through frameshift mutations causing insertions or deletions [5,12,15], while multiplex editing simultaneously targets multiple genomic sites using several guide RNAs within a single editing system [12,15]. In contrast, base editing achieves the direct, DSB-free conversion of one DNA base pair into another [5,12,15] and prime editing does not need a double-strand break (DSB) or a donor DNA template because it functions like a “search-and-replace” tool, allowing researchers to insert, delete, or substitute specific pieces of DNA directly at the target site [5,16]. The technology has also evolved into advanced insertion platforms, such as CRISPR-transposase systems, which enable the insertion of large DNA sequences beyond the size limits of standard prime editing [17]. Beyond single-locus precision, these tools also enable de novo domestication, the rapid introduction of domestication-associated traits into wild or semi-domesticated plants in place of generations of conventional selective breeding [12,16], as well as fine-tuning of gene expression through CRISPRa (CRISPR activation) and CRISPRi (CRISPR interference), which use a Cas protein that can no longer cut DNA and is guided to a gene’s regulatory region, where it turns the gene’s expression up or down without changing the underlying DNA sequence itself [5,12]. Apart from genome editing, CRISPR/Cas systems have also been repurposed for pathogen diagnostics: Specific High-sensitivity Enzymatic Reporter unLOCKing (SHERLOCK), for instance, is a CRISPR/Cas13a-based nucleic acid detection technique that combines isothermal RNA amplification (RPA) with a target-activated, collaterally cleaving Cas13a enzyme which releases a visible fluorescent signal from a reporter probe only when the specific target sequence is present [18].

Generally, CRISPR technology excels because of its direct retargeting with sgRNA modification, diverse Cas variants and vast delivery compatibility. Nevertheless, it is important to state that often the choice of genome-editing technology used depends on the specific application and conditions [8].

**Figure 1:**
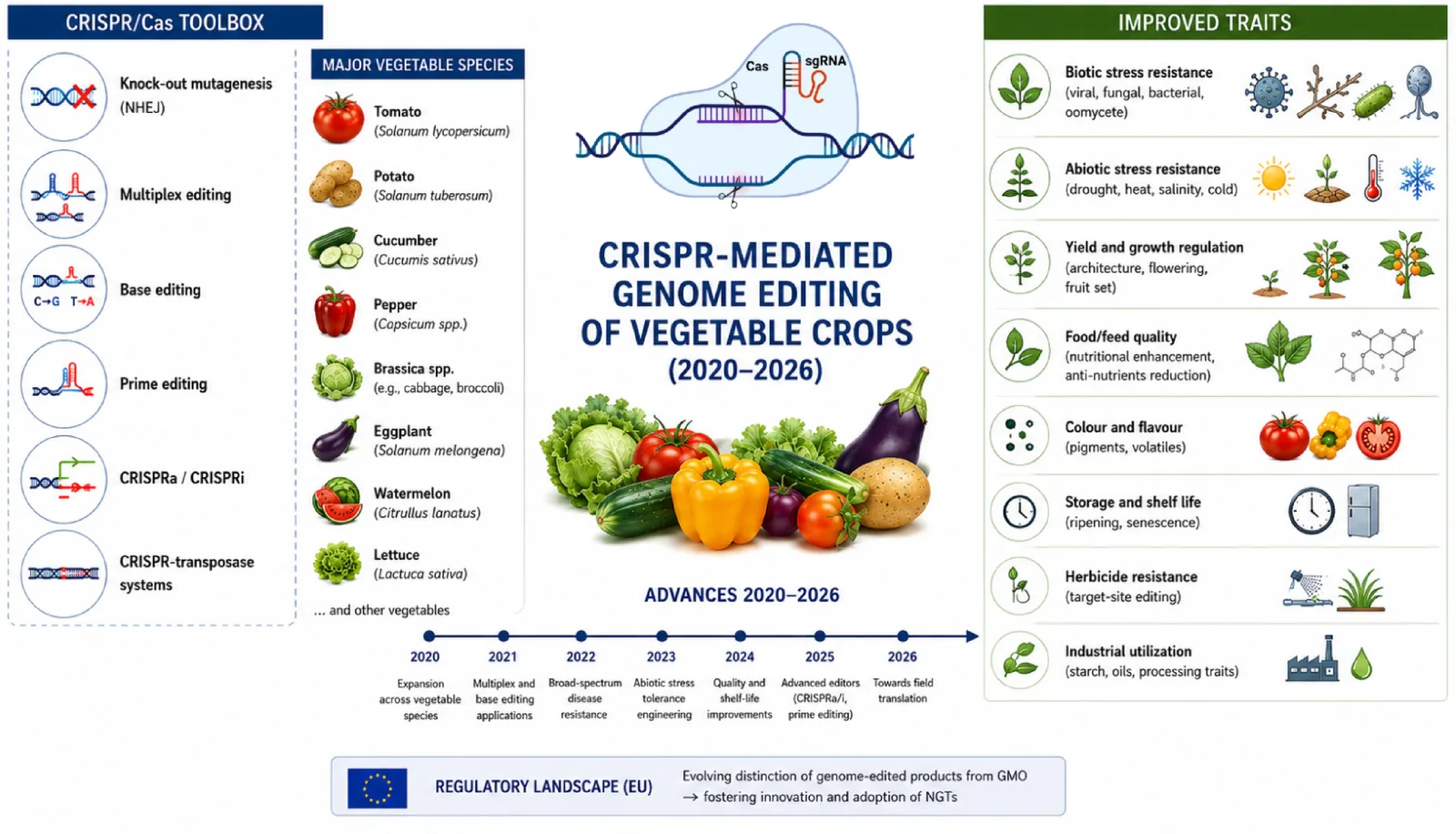
Overview of CRISPR/Cas-mediated genome editing in vegetable crops to date, highlighting the main species studied, dominant editing tools, key trait categories and emerging applications for precision vegetable breeding.

In vegetable crops, tomato and potato have drawn the most research interest in CRISPR/Cas editing, as they are among the most economically important crops globally and have well-established transformation protocols. This review examined multiple CRISPR-edited vegetable crops from 2020 to 2026 and drew conclusions regarding the key trait categories targeted, the crops most frequently studied, and the technologies employed (Figure 1). This review also discusses the regulatory environment and future perspectives for the field.

## 2. Breeding Objectives

### 2.1. Resistance to Biotic Stress

Biotic stress can cause enormous economic and food security problems because of pathogen-induced yield losses. Therefore, biotic stress resistance is the most actively pursued trait category in vegetable genome editing [2,3,19]. Generally, the goal is to target susceptibility (*S*) genes, which are host factors that pathogens require to establish infection [20,21]. Disruption of these host–pathogen interactions, without the introduction of foreign DNA, aims to create loss-of-function mutations in *S* genes, and that will generate heritable, and potentially durable, resistance [8,13]. Here, we cover a variety of viral, fungal, oomycete, bacterial, nematode and insect resistance types across multiple vegetable crops (Table 2).

#### 2.1.1. Viral Resistance

The existence of viruses depends on their hosts. Viruses have small, but complex, genomes, are highly adaptable and, in order to complete their infection cycle, harness and modify the resources of the host [22].

In agriculture, the use of chemicals for the containment of viruses is often ineffective and economically damaging for growers. Thus, breeding and development of disease-resistant crops is crucial for sustainable agriculture. The eukaryotic translation initiation factor 4E (eIF4E) is a host susceptibility factor required by many viruses to complete their replication cycle, and loss-of-function mutations in eIF4E genes confer recessive resistance. By CRISPR/Cas9-mediated editing of *eIF4E1* in tomato, resistance was simultaneously conferred to both potato virus Y (PVY) and cucumber mosaic virus (CMV) [23], dual editing of *SleIF4E1* and *SleIF4E2* conferred resistance to PVY [24], while editing of *eIF4E2* in cherry tomato provided resistance to pepper veinal mottle virus (PVMV) [25]. Tomato yellow leaf curl virus (TYLCV) is the most damaging disease of tomato crops [26]. Therefore, Faal et al. [27] employed the CRISPR/Cas9 system to produce TYLCV-resistant varieties by using a promoter driving the Cas9 that suppresses TYLCV. Additionally, Pramanik et al. [28] carried out the knockout of the *SlPelo* gene, a susceptibility gene for TYLCV, which successfully restricted the viral DNA proliferation, suppressed virus accumulation and limited the spread to non-inoculated plant parts, all while editing the *SlMlo1* gene, for powdery mildew resistance. Kravchik et al. [29] used CRISPR/Cas9 to generate single, double, and triple mutants of the tobamovirus susceptibility *SlTOM* family (*SlTOM1a*, *SlTOM3*, *SlTOM1b*). Simultaneous knockout of *SlTOM1a* and *SlTOM3* genes, which are essential for the replication of tomato brown rugose fruit virus (ToBRFV), generated tomato plants that were resistant to ToBRFV infection, while remaining fully susceptible to tobacco mosaic virus (TMV) and tomato mosaic virus (ToMV). Similarly, Ishikawa et al. [30] generated tomato plants with strong resistance to tobamoviruses, including ToBRFV, by multiplex CRISPR editing of *TOM1*. In cucumber, editing of *eIF4E* generated viral resistant cultivars of cucumber to watermelon mosaic virus (WMV), zucchini yellow mosaic virus (ZYMV) and papaya ringspot virus (PRSV) [31], while in watermelon, editing of *CleIF4E1* provided resistance to ZYMV [32]. In Chinese cabbage, editing of *eIF(iso)4E* increased resistance to turnip mosaic virus (TuMV) and revealed that CRISPR/Cas9 editing can accelerate the breeding process to improve traits [33]. In potato, PVY resistance has been conferred either by CRISPR/Cas9 editing of the *eIF4E1* gene [34] or by RNA targeting [35,36]. More specifically, broad-spectrum resistance was conferred through two distinct strategies: Noureen et al. [35] targeted multiple genes (*PI*, *HC-Pro*, *P3*, *Cl1*, *Cl2*, *VPg*) of PVY using CRISPR/Cas13 to achieve resistance against multiple PVY strains, while Zhan et al. [36] employed Cas13d to target PVY, potato virus S (PVS), potato virus X (PVX), and potato leafroll virus (PLRV), generating plants resistant to individual viruses or combinations of two to three simultaneously. In sweet potato, the CRISPR/Cas13 system targeted RNA to confer resistance against sweet potato virus disease (SPVD) [37]. Beyond direct resistance engineering, CRISPR has also been employed as a rapid and accurate diagnostic tool for plant virus detection. Specifically, the CRISPR/Cas12a method has allowed the differentiation of four major tobamoviruses: pepper mild mottle virus (PMMoV), ToBRFV, ToMV, and tomato mottle mosaic virus (ToMMV) [38]. Detection of tomato spotted wilt virus (TSWV) in tomato has been made possible by combining loop-mediated isothermal amplification (LAMP), a nucleic acid amplification technique that rapidly amplifies a specific target at a constant temperature, and CRISPR/Cas12a [39], and two geminiviruses (TYLCV, tomato leaf curl New Delhi virus; ToLCNDV) have also been detected using a LAMP/Cas12a platform [40]. Additionally, pospiviroids have been detected using a Cas13-based SHERLOCK approach [18].

#### 2.1.2. Fungal and Oomycete Resistance

Powdery mildew is among the most widespread fungal diseases of crops. The *MLO* (*Mildew Locus O*) gene family encodes proteins that are involved in the interaction between tomato and powdery mildew fungal pathogens and is the most widely targeted S-gene family to achieve powdery mildew resistance. In tomato, CRISPR/Cas9-mediated knockout of *SlMlo1* conferred complete resistance to *Oidium neolycopersici* (*On*) [28,41]. Santillán Martínez et al. [42] successfully generated and characterized different knockout events in the tomato ortholog of the *PMR4* gene, the callose synthase gene that produces callose in response to biotic and abiotic stresses, revealing that powdery mildew resistance in pmr4 mutants is linked to cell death upon infection, likely from salicylic acid pathway activation. Li et al. [43] targeted *DND1*, which encodes a cyclic nucleotide-gated cation channel (CNGC2) that regulates Ca^2+^ signaling and suppresses constitutive defense, and produced plants with reduced susceptibility to *On*, while maintaining normal plant growth and yield. In cucumber, Shnaider et al. [44] developed a powdery mildew resistant cucumber cultivar using CRISPR/Cas9-mediated mutagenesis of *CsaMLO8* under semi-commercial growth conditions, while Tek et al. [45] showed that resistant cucumber cultivars with a strong pre-invasive defense can be generated by editing of *CsaMLO8* or with a post-invasive defense by creating *CsaMLO1*/*CsaMLO11* mutations. Additionally, a triple *CsMlo1*/*CsaMLO8*/*CsaMLO11* cucumber mutant produced stronger and broader resistance than single-gene edits [46].

*Botrytis cinerea* is a plant necrotrophic fungus that causes gray mold in vegetables and softening in fruits [47]. By inactivating the phospholipase C2 gene, *SlPLC2*, with CRISPR/Cas9, Perk et al. [48] produced resistant tomato plants with less ROS and an increase and reduction in (JA) and (SA)-response marker genes, while CRISPR/Cas9-mediated knockout of *ACET1a* and *ACET1b* genes by Jeon et al. [49] abolished falcarindiol production in tomato, confirming that this pathogen-responsive acetylenase gene cluster is required for resistance to both bacterial and fungal pathogens. Mutation of the ubiquitin E3 ligase gene *PUB17* in tomato reduced susceptibility to necrotrophic fungi including *Botrytis cinerea* and *Alternaria solani* [50]. Fusarium wilt resistance was achieved in tomato through editing of *DDTFR10/A* [51] and editing of both *XSP10* and *SlSAMT* against *Fusarium oxysporum* f. sp. *lycopersici* [52]. In potato, CRISPR/Cas9 knockout of *StDND1*, *StCHL1* and *StDMR6-1* conferred increased resistance to *Phytophthora infestans*, the most serious disease of potato crops worldwide [53]. Editing of *StPM1* improved *Phytophthora* resistance without affecting growth and development [54] and multiplex editing of *StDMR6-1* produced lines with increased resistance to *Phytophthora infestans*, *Alternaria solani* and common scab [55]. In eggplant, editing of *DMR6-1* enhanced resistance to *Phytophthora* spp. [56]. In Chinese kale, multiplex editing of BTB-POZ, MATH domains of the *BoBPM6* and *DMR6* genes in a single transformation event provided broad-spectrum resistance against fusarium wilt, black rot and clubroot [57]. In watermelon, knockout of *ClPSK1* enhanced resistance to *Fusarium oxysporum* f. sp. *niveum* [58]. In chili pepper, *CaERF28* knockout produced T-DNA-free lines with enhanced resistance to *Colletotrichum truncatum* (anthracnose) and improved expression of defense responsive genes [59]. In cucumber, research from Dong et al. [60] revealed a target gene that is elite for breeding varieties with broad-spectrum stress tolerance. Specifically, they discovered an SNP in the *CsSGR* coding region and produced knockout mutants with tolerance to low temperature, salinity, water deficit and resistance to *Pseudoperonospora cubensis*. Moreover, editing of the DMR6 gene family has led to the production of Sldmr6-1 mutants in tomatoes that conferred broad-spectrum disease resistance against bacteria (*P. syringae*, *X. gardneri*, *X. perforans*), oomycete (*P. capsici*) and fungi (*P. neolycopersici*) [61]. DMR6-1 is also an interesting multi-stress breeding target for simultaneously improving resistance to *Phytophthora infestans* and acquiring drought tolerance [62].

#### 2.1.3. Bacterial, Nematode and Insect Resistance

Editing of *SlBs5* and *SlBs5L* in tomato conferred resistance to *Xanthomonas perforans* bacterial spot, which is a devastating disease for both tomato and pepper [63], while knockout of *SlNRX1*, which is negatively related with plant immunity, improved resistance to both *Pseudomonas syringae* pv. *maculicola* and *Alternaria brassicicola* [64]. Without affecting crop productivity, García-Murillo et al. [65] employed the CRISPRa system to epigenetically edit tomato plants by editing *SlPR-1* to provide enhanced resistance to *Clavibacter michiganensis* subsp. *michiganensis*. Leibman-Markus et al. [66] edited *SlNRC4* and gained increased basal immunity and broad-spectrum disease resistance. In potato, knockout of *NPR3* enhanced resistance to *Candidatus* Liberibacter spp., which causes significant economic losses globally [67]. In tomato, *SlZRK1* knockout resulted in resistance to herbivorous insects by increasing the accumulation of jasmonic acid [68], and editing of *ARF8A* and *ARF8B* decreased susceptibility against root-knot nematodes [69]. Finally, CRISPR/LbCas12a, a visual detection method, was applied to detect bacterial fruit blotch pathogens in watermelon, with *Acidovorax citrulli* being the main one [70], illustrating the dual role of CRISPR as both an editing tool and disease diagnostic platform.

**Table 2:** Selected CRISPR/Cas-mediated biotic stress resistance targets in vegetable crops (2020–2026).

Pathogen	Crop	Target Gene(s)	CRISPR/System	Reference
TYLCV (virus)	Tomato	Viral genome (*IR*, *CP*)	Cas9	[27]
TYLCV and powdery mildew	Tomato	*SlPelo*, *SlMlo1*	Cas9	[28]
Tobamovirus (ToBRFV)	Tomato	*SlTOM1*, *SlTOM3*	Cas9	[29,30]
PVY and CMV (potyvirus)	Tomato	*eIF4E1*	Cas9	[23]
WMV, ZYMV, PRSV	Cucumber	*eIF4E*	Cas9	[31]
TuMV (potyvirus)	Chinese cabbage	*eIF(iso)4E*	Cas9	[33]
PVY (multiple strains)	Potato	*eIF4E1*, *CP*, *PI*, *HC-Pro*, *P3*, *Cl1*, *Cl2*, *VPg*	Cas9/Cas13	[34,35,36]
ZYMV	Watermelon	*CleIF4E1*	Cas9	[32]
Powdery mildew (*Oidium*)	Tomato	*SlMlo1*, *PMR4*, *DND1*	Cas9	[28,42,43]
Powdery mildew	Cucumber	*CsaMLO8*, *CsMlo1/8/11*	Cas9	[44,46]
*Botrytis cinerea* (grey mold)	Tomato	*SlPLC2*, *DACET1*, *D270*, *PUB17*	Cas9	[48,49,50]
*Phytophthora infestans* (late blight)	Potato	*StDND1*, *StCHL1*, *StDMR6-1*, *StPM1*	Cas9	[53,54,55]
*Phytophthora* spp.	Eggplant	*DMR6-1*	Cas9	[56]
*Fusarium wilt* (FON)	Watermelon	*ClPSK1*	Cas9	[58]
*Fusarium wilt*, black rot, clubroot	Chinese kale	*BoBPM6*, *DMR6*	Cas9	[57]
Anthracnose (*Colletotrichum*)	Chilli pepper	*CaERF28*	Cas9	[59]
Multi-pathogen broad spectrum	Cucumber	*CsSGR*	Cas9	[60]
*P. syringae*, *Phytophthora*, *Xanthomonas*	Tomato	*SlDMR6-1*, *SlDMR6-2*	Cas9	[61]
*Xanthomonas* (bacterial spot)	Tomato	*SlBs5*, *SlBs5L*	Cas9	[63]
Root-knot nematode	Tomato	*ARF8A*, *ARF8B*	Cas9	[69]
Aphids (insect)	Watermelon	*ClVST1*	Cas9	[71]

### 2.2. Resistance to Abiotic Stress

Abiotic stress tolerance is increasingly prioritized as climate change intensifies drought, heat, and salinity events across vegetable-growing regions. Due to global warming and the increasing world population, there is a growing need to produce high-yielding cultivars with improved tolerance to abiotic stresses (Table 3).

#### 2.2.1. Drought Stress

Drought stress is the greatest threat to global food security [72] and one of the most significant challenges for tomato production [73]. In order to enhance stomatal regulation and water-use efficiency, genes, such as *GID1* and *SLY1* [74], *SlLBD40* [75], *SlUDPGT* [76] and *SlGT30* [77], have been edited. *SlPAO3* editing improved drought resistance through polyamine oxidase regulation, by diminishing xylem hydraulic conductivity that limits water loss and reducing vulnerability to embolism [78], while *SlDMR6-1* editing led to drought-resistant mutants [62]. In Chinese cabbage, *PRR1a* and *PRR1b* editing regulated the circadian clock to improve adaptation to high temperature and drought conditions caused by global warming [79]. Finally, Sohail et al. [80] edited *CmoPIP1-4*, which enhances drought resistance in yeast, and Shen et al. [81] performed *SlALKBH10B* knockout, which enhanced both drought and salt stress tolerance.

#### 2.2.2. Heat Stress

Heat stress tolerance was addressed in tomato by *BAG9* knockout, which confers thermotolerance and the stability of heat-shock proteins [82], and *CPK28* knockout also influenced thermotolerance [83]. Notably, a prime editing (PE) application achieved knock-in of a heat-shock element in the cell-wall-invertase gene (*CWIN*) and improved heat resilience [84], which demonstrated the possibility of performing a precision insertion, rather than a simple knockout, for abiotic stress engineering.

#### 2.2.3. Salt Stress

To acquire salt stress tolerance, genes such as *SlHyPRP1* [85,86], *HKT1*,*2* [87], *SlARF4* [88], have been edited, *SlSTOP1* and *SlSZP1* for aluminum resistance [89] and *SlHAK20* for salt stress [90]. In cucumber, *CsHAK5;3* mediates K^+^ absorption and plays a vital role in acquiring salt tolerance [91]. Additionally, in *Cucurbita moschata*, Sohail et al. [92] identified 21 plasma membrane intrinsic proteins (*PIPs*), genome-wide, which are important for salinity and drought tolerance in plants. They further produced knockout *CmoPIP1-4* mutants to functionally characterize the genes and by overexpressing *CmoPIP1-4* they conferred salinity tolerance to yeast. Multiple CRISPR studies targeted salinity tolerance through overexpression of *CmRbohD1* and *CmRbohD2* in pumpkin plants [93], while Wei et al. [94] showed that *CmCNIH1*, which influences the trafficking of *CmHKT1;1*, improved salt tolerance. Geng et al. [95] explored salt tolerance mechanisms using a cucumber/pumpkin graft, by editing the sodium transporter gene *CmoHKT1;1* and by overexpressing of pumpkin tonoplast Na+/H+ antiporter gene *CmoNHX4*. Finally, Peng et al. [96] revealed that in pumpkin rootstocks *CmoNAC1* bound to the promoters of *CmoAKT1;2* and *CmoHKT1;1*, increased the K+/Na+ ratio, and improved salt tolerance of grafted cucumbers.

#### 2.2.4. Cold, Chilling and High Light Stress

Cold stress inhibits plant growth and development and reduces productivity. Therefore, breeding cultivars with enhanced cold tolerance is crucial. For that reason, Fan et al. [97] reported that the *BAM* gene *SlBAM3* plays a critical role in tomato cold tolerance, and via CRISPR/Cas9 editing they generated knockout mutants with reduced starch degradation and sugar accumulation. In potato, *VInv* knockout could increase resistance to cold stress [98]. Knockout of BZR1-interacting WD40-repeat protein (BIW) conferred chilling tolerance in tomato [99], and *StAOX* editing addressed high light stress through regulation of cyanide-resistant respiration [100].

**Table 3:** Selected CRISPR/Cas-mediated abiotic stress resistance targets in vegetable crops (2020–2026).

Abiotic Factor	Crop	Target Gene(s)	CRISPR/System	Reference
Salt/osmotic stress	Tomato	*SlARF4*	Cas9	[88]
Salt stress	Tomato	*SlHyPRP1*	Cas9	[85]
Salt stress	Tomato	*HKT1*,*2*	LbCpf1-geminiviral	[87]
Salt/osmotic stress	Tomato	*SlHAK20*	Cas9	[90]
Drought	Tomato	*GID1*, *SLY1*	Cas9	[74]
Drought	Tomato	*SlLBD40*	Cas9	[75]
Drought	Tomato	*SlUDPGT*	Cas9	[76]
Drought/salt stress	Tomato	*SlALKBH10B*	Cas9	[81]
Drought	Tomato	*SlGT30*	Cas9	[77]
Drought	Tomato	*SlPAO3*	Cas9	[78]
Drought	Tomato	*SlDMR6-1*	Cas9	[62]
Heat stress	Tomato	*BAG9*	Cas9	[82]
Heat stress	Tomato	*CPK28*	Cas9	[83]
Heat resilience	Tomato	*CWIN*	Cas9 [Prime Editing]	[84]
Cold stress	Tomato	*SlBAM3*	Cas9	[97]
Aluminum toxicity	Tomato	*SlSTOP1*, *SlSZP1*	Cas9	[89]
Heavy metal/heat	Tomato	*SlGRXS14*, *SlGRXS15*, *SlGRXS16*, *SlGRXS17*	Cas9	[101]
UV-B/high light	Tomato	*SlUVR8*	Cas9	[102]
Resilience/productivity	Tomato	*BIW*	Cas9	[99]
Cold stress	Potato	*VInv*	Cas9	[98]
High light stress	Potato	*StAOX*	Cas9	[100]
Heat stress (parthenocarpy)	Pepper	*CaPAD1*	Cas9	[103]
Broad-spectrum (low temp/salt/drought/ downy mildew)	Cucumber	*CsSGR*	Cas9	[60]
Salinity	Cucumber	*CsHAK5;3*	Cas9	[91]
Salt/drought	Cucurbita moschata	*CmoPIP1;4*	Cas9	[80,92]
Salinity	Cucurbita moschata	*CmoHKT1;1*	Cas9	[95]
Salinity	Cucurbita moschata	*CmoNAC1*, *CmoNCED6*	Cas9	[96]
Salinity	Cucurbita moschata	*CmoDREB2A*	Cas9	[104]
Salinity	Cucurbita moschata	*CmRbohD1/D2*, *CmCNIH1*	Cas9	[93,94]
High temperature/ circadian rhythm	Chinese cabbage	*PRR1a*, *PRR1b*	Cas9	[79]

### 2.3. Yield, Plant Architecture and Growth Regulation

Plant architecture profoundly influences harvestable yield, ease of mechanized harvesting, and suitability for controlled environment agriculture.

In tomato, crop yield can be improved, by producing dwarf plants through editing of *YABBY2b* [105] and *SlYTH2* [106], and simultaneous editing of *DWARF* (*D*) and *SELF-PRUNING* (*SP*) genes [107]. Editing of *SlER, SP5G and SlER* generated compact tomato cultivars with decreased growth cycles, which are suitable for urban agriculture [108], and compact growth with early fruit production was achieved through base editing of *SUPPRESSOR OF SP2* (*SSP2*) [109].

Simultaneous knockout of *SlKIX8* and *SlKIX9* produced enlarged leaves and fruits with increased pericarp thickness [110]. Editing of *SlDOF9*, an auxin-responsive transcription factor, increased flower and fruit number [111], while *SlTPL3* editing generated fruits with more locules [112]. At the meristem level, *ENO* editing controlled meristem size to increase fruit yield [113]. Finally, *SlHB8* editing, which targets a negative regulator of tapetum development, increased pollen activity and improved fruit set [114].

Early flowering was achieved through *SlFAF* family editing [115]. *JOINTLESS2* editing could create a shortened plant architecture with a jointless pedicel, allowing ground cultivation systems that do not require the support of stakes and ties and could be suitable for mechanical harvesting [116].

In lettuce, Qi et al. [117] targeted delayed bolting, a key trait for prolonging the quality and marketability of the crop, through editing of *LsKN1* and *LsOFP6*. Choi et al. [118] edited *LsSOC1* and increased leaf yield by delaying the onset of flowering. Beracochea et al. [119] edited *SPL13* to delay the appearance of flower buds and achieve increased yield, and An et al. [120] edited *LsNRL4* to improve photosynthesis and plant architecture. In Chinese cabbage, *BrLEAFY* and *AGAMOUS-like* editing delayed bolting [121,122], while *BraRGL1* editing promoted early bolting and flowering and can be a target for the breeding of early maturing varieties [123].

In asparagus and lettuce, *aspSPL14* editing produced dwarf phenotypes to increase yield [124]. In potato, *FtsZ1* editing increased the size of starch granules, which is a key parameter for industrial processing [125], and *StDRO2* editing increased plant height, root depth and tuber weight [126]. In cucumber, knockout of *CsHT16* revealed its role in maintaining sugar homeostasis, and the collective role of *CsHT3*, *CsHT12* and *CsHT16* in regulating the supply of carbohydrates for fruit enlargement [127], and *CSa2G264590* knockout increased spine density [128]. In eggplant, *PPO2* editing produced multiple agronomically important phenotypic changes including plant dwarfing, early flowering and early fruit set, thus revealing the hidden pleiotropy of the *PPO* gene [129].

### 2.4. Improvement of Food/Feed Quality

Improving the nutritional profile, flavor and processing quality of vegetable crops is very important for consumer-focused breeding.

In tomato, editing of *SlINVINH1* and *SlVPE5* enhanced soluble sugar content, which increases sweetness and tomato sauce yield [130]; *SlINVINH1* increased sugar content, one of the most important quality traits of tomato [131]; *SlbZIP1* increased sugar and amino acid content [132]; and *SlVIF* increased the activity of the *VIN* enzyme, fructose and glucose and decreased sucrose content, resulting in delayed fruit ripening and extended shelf-life [133]. Hunziker et al. [134] applied Target-AID technology for generation of substitution mutations in three genes (*SlDDB1*, *SlDET1*, *SlCYC-B*), which are responsible for carotenoid accumulation. Editing of *SlARF5*, *SlARF7*, *SlARF8A* and *SlARF8B* produced parthenocarpic fruits that are preferred by consumers [135], and editing of *SlSPL-CNR* increased the content of flavonoids, which play a role in fruit color and are important for human health [136]. The content of vitamin D3 was boosted through editing of *Sl7-DR2* [137] and heavy metal cadmium accumulation was reduced through *Sl1* editing [138].

In potato, some of the edits include *GBSSI* knockout to produce amylose-free waxy starch in tubers for food processing [139,140]. In addition, mutations were created in all eight *Sbe* alleles to develop potato starch with no detectable branching. In food products, high amylose content and long amylopectin chains contribute to a low glycemic index (GI) after intake and have health benefits [141]. *StSSR2* knockout reduced the content of steroidal glycoalkaloids (SGAs), which can be toxic to humans and damage the quality of potato tubers [142]. *StISAC* editing increased anthocyanin accumulation [143], *StbHLH47* mutants exhibited increased iron content in the potato plants [144], and *StGGP* editing elevated vitamin C content in potato tubers [145]. Lastly, through *VInv* and *AS1* editing, Ly et al. [146] and Yasmeen et al. [147] reduced the production of acrylamide, a contaminant, potential carcinogen and neurotoxin, and improved cold storage traits. In broccoli, Kim et al. [148] utilized the CRISPR/Cas9 system to mimic a naturally occurring *MYB28* allele with a 9 bp deletion in exon 3, which is associated with elevated glucoraphanin accumulation. This causes a single amino acid substitution and the loss of three amino acids and is already found in the wild species *Brassica villosa*. By editing MYB28 transcription factor, one of the key regulators involved in the accumulation of glucosinolate levels in broccoli, they produced plants enriched with glucoraphanin, a major glucosinolate, which protects the human body against several chronic diseases. In Chinese kale, *BoaAOP2* editing enhances glucoraphanin levels, and its hydrolysis product has powerful anticancer activity [149]. In cucumber, *CsHEC2* editing boosted cytokinin biosynthesis and elevated fruit wart formation, an important quality trait that greatly affects market value and fruit appearance [150]. In chicory, *CYP71DD33* editing led to accumulation of anti-inflammatory 8-deoxylactucin and its derivatives [151]. In lettuce, editing of *LCY-ε*, *uGGP1*, *uGGP2* and *CCD4*, enhanced ascorbic acid, β-carotene and zeaxanthin for improved nutritional value [152].

### 2.5. Improvement of Color/Flavor

Color and flavor traits directly influence consumer preference and market value. Fruit color is an important trait that affects consumer preferences and in tomatoes it is determined by different pigment accumulation, such as carotenoids, flavonoids and chlorophyll. CRISPR/Cas9 has been used to generate a wide variety of fruit colors through editing of *PSY1*, *MYB12* and *SGR1* [153], while a tangerine color was achieved through editing of the *CRTISO* locus [154]. In order to identify the sensorial and chemical components that determine fruit flavor variation, by validating a QTL that affects the levels of the phenylalanine-derived volatiles (PHEVs), Tikunov et al. [155] performed CRISPR/Cas9-mediated editing, as a reverse-genetics approach, and revealed that *FLORAL4* was the key factor that affects PHEV accumulation. *Sl-AKR9* knockout impacted phenylacetaldehyde, glucose and fructose content, which are major contributors to flavor in many foods [156]. *SlVIF* knockout increased fructose and glucose, but decreased sucrose, thus altering flavor and extending shelf-life [133]. *SlYTH2* knockout enriched fruit aroma [157].

In carrot, *DcBCH1* editing changed taproot color from orange to pink-orange [158], and knockout of *DcMYB11c* in purple taproot carrot with purple petioles produced a pale purple phenotype, due to the dramatic decrease in anthocyanin content [159]. In Chinese kale, editing of *BoaCRTISO* produced yellow-colored stems and leaves [160,161], while editing of *BocPDS1* and *BocPDS2* produced albino phenotypes [162]. In brown mustard, editing of the type-I myrosinase multigene family reduced pungency by disarming the “mustard bomb” reaction that is characterized by an unpleasant flavor and/or odor in leafy *Brassica* [163]. In watermelon, *ClVST1* overexpression increased sucrose content [164]. Finally, albino phenotypes have been generated through *PDS* editing and have been confirmed across numerous vegetable species, serving as proof-of-concept.

### 2.6. Post-Harvest Quality

Postharvest quality and shelf-life are critical determinants of food security and commercial value. In tomato, *SlNAC4* editing altered fruit ripening and softening, two key traits for fleshy fruit [165]. Double knockout of *SlPG2a* and *SlPL*, two pectin-degrading enzymes, improved tomato shelf-life, while ensuring fruit quality [166]. *TAGL1* editing delayed the onset of ripening [167]; *SlNAP1* editing delayed color change [168]; *SlXTH5* editing improved fruit firmness [169]; and *SPL-CNR* editing could enhance the storage potential of ripening fruits and is a potential target for modulating ethylene, fruit softening and flavor-related volatiles in tomato [170]. *RIN* knockout delayed the onset of ripening [171].

In potato, knockout of *StPPO2* reduced enzymatic browning [172], suppression of *StPHB3* reduced browning of freshly cut potatoes by enhancing the antioxidant activity and inhibiting the activation of *PPO* [173], editing of starch phosphorylase genes decreased cold-induced sweetening of potato tubers [174] and *SBE3* editing by the CRISPR/dMac3-Cas9 system regulated amylose content [175]. In eggplant, simultaneous knockout of *SmelPPO4*, *SmelPPO5* and *SmelPPO6* reduced fruit flesh browning after cutting [176]. In melon, *CmACO1* knockout produced fruits that remained green and had higher firmness and no early fermentation, resulting in extended shelf-life [177].

### 2.7. Herbicide Resistance and Industrial Utilization

Herbicide tolerance enables efficient weed management and has been conferred through CRISPR base editing and point mutation strategies across several vegetable crops.

In tomato, *ALS* editing conferred chlorsulfuron resistance [178], while editing-efficiency testing of sgRNAs targeting *PDS*, *ALS*, and *EPSPS* provided an indication of the editing efficiency that could subsequently be achieved during the transformation process for the same trait [179]. In potato, prime editing of *StALS* achieved herbicide chlorsulfuron resistance [180]. In cauliflower, editing of *ALS* and *CENH3* produced *ALS*-herbicide resistance [181].

Industrial utilization studies involve editing to obtain male sterility and improve self-compatibility. In tomato, multiple CRISPR studies generated male sterility lines (MLS) through editing of *SlPHD_MS1* [182], *SlAMS* [183], *SlACO2* [184], *SlSTR1* [185], *SlMS10* [186], *Ms1035* and *GSTAA* [187] and *SlDYT1* and *SlGSTAA* [188]. Increased crossover frequency was achieved in tomato through *RECQ4* editing, as crossover formation during meiosis is essential for the introduction of favorable alleles [189]. In potato, knockout of *S-RNase* and *Sli* generated self-compatible diploid lines for efficient hybrid breeding [190,191]. In watermelon, editing of *ClATM1* caused male sterility [192] and editing of *ClDMP4* established maternal haploid induction for doubled haploid production [193]. In Chinese cabbage, *BrVRN1* was edited to control flowering time for breeding purposes [194].

## 3. Editing Key Vegetable Crops

In vegetable crops, the development and application of genome editing in tomato and potato is being studied the most, probably due to their economic importance, wide availability of genomic resources, and efficient tissue culture, as well as transformation via Agrobacterium [195].

### 3.1. Tomato (Solanum lycopersicum)

**Figure 2:**
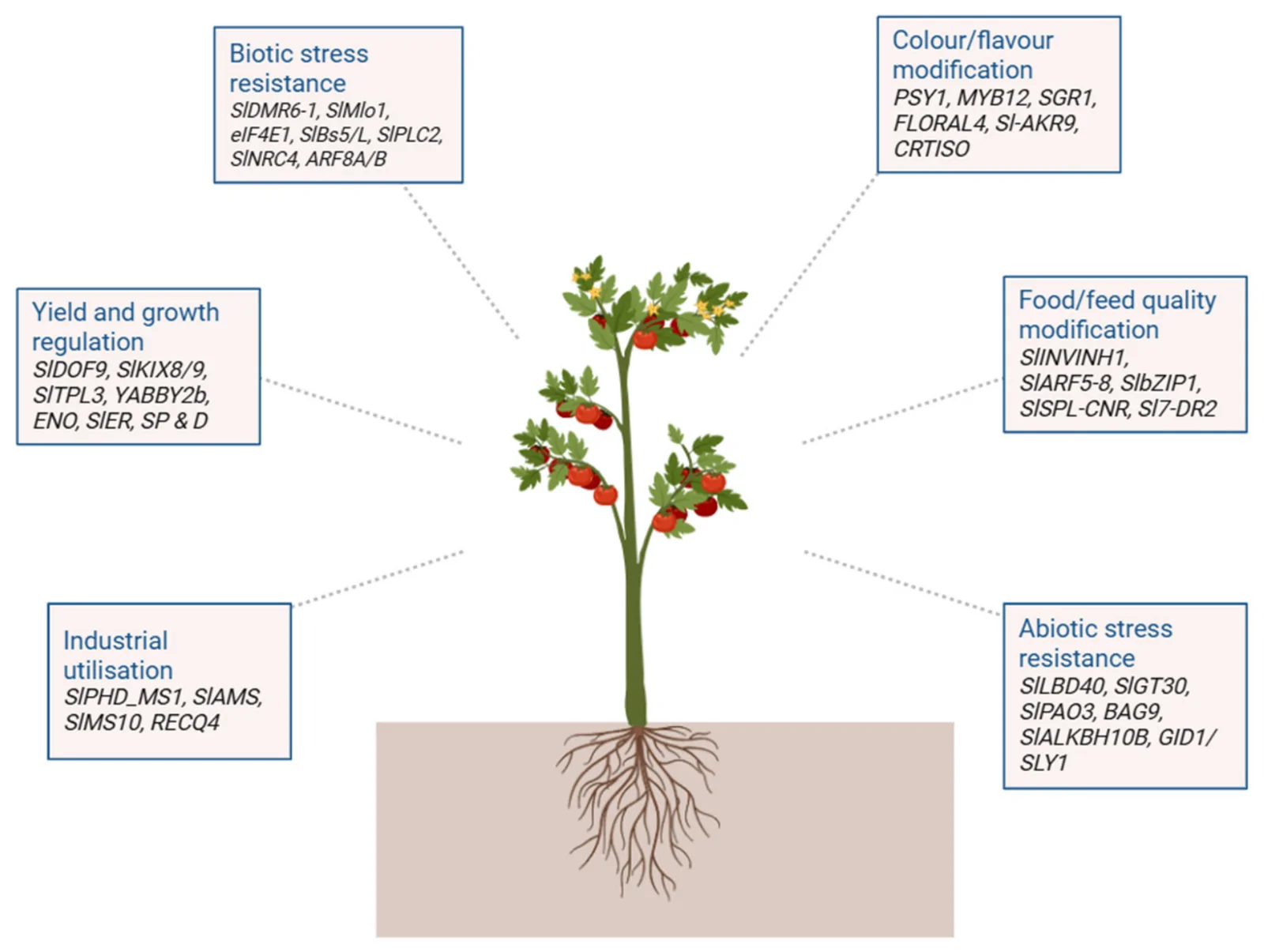
Edited trait categories and target genes in tomato based on genome-editing studies published between 2020 and 2026.

Tomato is the most widely grown vegetable crop [196], is one of the most economically important vegetables globally and is consumed both fresh and processed [197]. In this review we confirmed that tomato is the most widely studied vegetable regarding CRISPR research for the years 2020–2026 (Figure 2). The three largest trait categories that draw both interest and research are biotic stress resistance, yield and growth regulation, and abiotic stress resistance.

Among biotic stresses, viral and fungal pathogens represent the primary targets. *TYLCV*, in particular, is the most damaging viral disease in tomato crops [26] and powdery mildew (*Oidium neolycopersici*), a fungal disease, affects the productivity of the crop [43]. A particularly interesting case is the single editing of the *SlDMR6-1* gene by Thomazella et al. [61], which conferred resistance across three pathogen species simultaneously. For quality improvement, some commercially desired traits are parthenocarpy, increased sugar content, lycopene accumulation and improved shelf-life. Additionally, simultaneous editing of flowering-time and fruit-set regulators can create compact, early-bearing, day-neutral plants within a single generation, which is important for the adaptation of tomatoes under controlled conditions [107,108]. For abiotic stresses, drought is the dominant focus, due to global warming and the increasing world population, and drought-tolerant plants are successfully obtained via multiple ABA pathways and transcription factor gene editing.

### 3.2. Potato (Solanum tuberosum)

**Figure 3:**
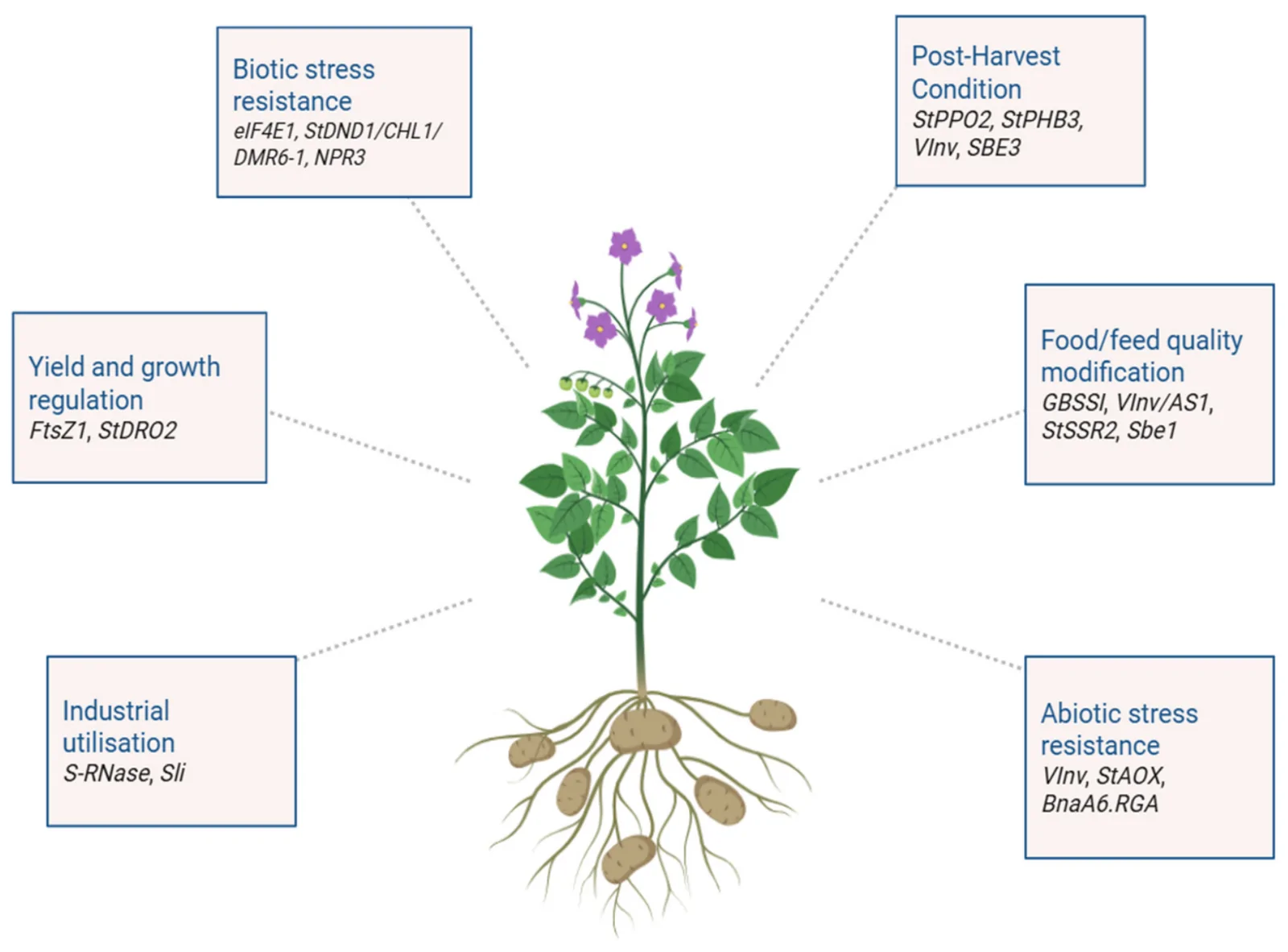
Edited trait categories and target genes in potato based on genome-editing studies published between 2020 and 2026.

Potato is the fourth most important food crop in the world, after rice, maize and wheat [198]. This staple crop presents challenges for genome editing as a tetraploid, highly heterozygous and clonally propagated crop. For successful editing, all alleles at a given locus must be disrupted simultaneously, to acquire a homozygous genotype, which is made more efficient by applying CRISPR multiplex editing [198,199]. In this review we confirmed that potato is the second most widely studied vegetable regarding CRISPR research for the years 2020–2026 (Figure 3), with biotic stress resistance, food/feed quality modification and storage conditions representing the largest categories.

Editing for biotic stress resistance has drawn most of the research interest. Potato virus Y (PVY) is devastating for the crop, as it affects the tuber quality and yield, with crop losses recorded to surpass 80% [200]. CRISPR is being used to increase resistance to PVY in potatoes [34,35,36]. Another threat to potato production worldwide is *Alternaria solani*, a fungus and the cause of early blight in potato. With CRISPR it could be visually detected [201] and crop resistance could be acquired [55]. Lastly, the fungus *Phytophthora infestans*, the causal agent of late blight, affects the growth rate and quality of potato tubers. Infected crops can experience yield losses of up to 80%, and in low-income and developing countries it can be particularly devastating by causing tuber rot and threatening food security [202]. Hence, several studies aim to increase resistance to the fungus [53,54,55].

CRISPR has also been applied to regulate food and/or feed quality in potatoes. Efforts focus on eliminating the amylose component of starch, and increase the amylopectin level, which has many food processing and industrial applications [139,140]. Moreover, research by Zhang et al. [145] aimed to elevate vitamin C content in potato tubers through CRISPR editing of the upstream open reading frames (uORFs) of two *StGGP* genes that encode GDP-L-galactose phosphorylase. This strategy boosted the accumulation of ascorbate while also maintaining yield and nutrient balance, which further revealed that excessive vitamin C enrichment disrupts auxin signaling and incurs developmental penalties.

Editing for better storage conditions aimed to avoid the formation of acrylamide, a classified carcinogen, during storage of potato tubers at cold temperatures. By lowering the accumulation of reducing sugars, processed potatoes contain less acrylamide [146,147].

## 4. Focus on Cucurbit Crops

There is great interest from our part in CRISPR editing of crops belonging to the Cucurbitaceae family. Therefore, cucumber and watermelon were selected as representative cucurbit species, because they appear to be both the most edited and the most economically significant in the family.

### 4.1. Watermelon (Citrullus lanatus)

Watermelon is the most important cucurbit crop globally, with production for 2024 exceeding 100 million tons [203]. Despite its economic importance, CRISPR adoption has stayed behind, due to significant recalcitrance to Agrobacterium-mediated transformation. A high-quality genome sequence was made available in 2013 by Guo et al., and recent breakthroughs using chimeric morphogenic regulators (*GRF4-GIF1*) have substantially improved transformation efficiency [204].

In watermelon, research with CRISPR/Cas is mainly focused on conferring biotic stress resistance. The fungus *Fusarium oxysporum* f.sp. *niveum* is the causal pathogen of fusarium wilt and resistance was achieved with knockout of *ClPSK1*, a gene that encodes the phytosulfokine peptide precursor that suppresses plant immunity [58]. Aphid resistance was also achieved by disrupting *ClVST1*, and as a result insect colonization was reduced without visible agronomic consequences [71]. Regarding viral diseases, Li et al. [32] performed knockout of *CleIF4E1*, which encodes a eukaryotic translation initiation factor and acts as a susceptibility gene, to generate plants resistant to ZYMV, which affects crop quality and yield, and results in considerable economic losses. Additionally, CRISPR/Cas12a was used as a diagnostic tool by Wang et al. [70], who developed an amplification-free LbCas12a-based assay for the specific and sensitive detection of the seed-borne bacterial pathogen *Acidovorax citrulli*, the causal pathogen of bacterial fruit blotch.

Lastly, CRISPR/Cas has been used for other purposes, such as food quality improvement by increasing sucrose content by *ClVST1* editing [164] and decreasing seed size with *ClBG1* editing [205]. Male sterility was achieved, by editing *ClATM1* [192], and producing double haploid plants via maternal haploid induction by editing of *ClDMP4* [193]. Watermelon is an increasingly important CRISPR model for cucurbit crop improvement, especially as transformation protocols continue to improve.

### 4.2. Cucumber (Cucumis sativus)

Cucumber is the second most cultivated cucurbit crop globally, after watermelon [203], and has attracted considerable CRISPR attention, particularly for biotic stress resistance and fruit quality modification.

Powdery mildew, one of the most economically damaging diseases in cucumber production, has been targeted through editing of *MLO* genes. Shnaider et al. [44] performed single knockout of *CsaMLO8*, which conferred strong resistance under semi-commercial conditions, while Dong et al. [206] caused double knockout of *CsMLO8* and *CsMLO11*, therefore expanding resistance; the triple mutant *CsMlo1/8/11*, created by multiplex editing, achieved the most comprehensive protection to date [46]. Viral resistance against WMV, PRSV and ZYMV was achieved through knockout of the *eIF4E* gene, which is one of the target genes that plays a key role in viral infection during interaction with potyvirus viral proteins [31].

Dong et al. [60] performed knockout of *CsSGR* (*STAYGREEN*) to confer tolerance to low temperatures, salinity and water deficit, and simultaneously achieved partial resistance to downy mildew (*Pseudoperonospora cubensis*). Salinity tolerance was further addressed through editing of *CsHAK5;3*, a key player under saline conditions in K+ homeostasis in grafted cucumbers [91].

For the improvement of fruit quality, a commercially critical trait is wart formation. *CsHEC2* knockout reduced wart density [150] and *CsEMS1* editing reduced wart number and density [207], while *CsLrp1* editing significantly increased wart density [208].

Additional quality traits include increasing spine density through *Csa2G264590* knockout, as these varieties are popular in Europe and West Asia [128], and modifying fruit size and shape through *CsHT16* knockout [127]. Notably, Ni et al. [209] developed a biofortified cucumber with potential cardiovascular health benefits by combining the naturally low sugar content of cucumber with the reported cardiovascular benefits of nattokinase (NK), a serine protease which is secreted by *Bacillus subtilis* var. *natto*. By utilizing the CRISPR/Cas9 system, the researchers replaced the *Bitter fruit* (*Bt*) gene, which contributes to bitter taste with no obvious impact on growth and development, with NK. In this manner, they constructed a therapeutic cucumber that could express NK, thus preventing thrombosis, improving blood biochemical parameters and alleviating cardiovascular diseases by consumption.

## 5. Legislation and Commercialization of CRISPR-Edited Vegetable Crops

The global regulatory framework for genetically modified and genome-edited plants is based on the question of whether products that have undergone genome editing should be evaluated on the basis of the production process or on the characteristics of the final product [210]. This distinction is particularly significant given that editing without introducing foreign DNA into cells may mitigate regulatory concerns related to genetically modified organisms. CRISPR/Cas components can be delivered to plant cells through methods such as Agrobacterium-mediated transformation, which introduces foreign DNA that may remain in the genome, or through transgene-free approaches such as ribonucleoprotein (RNP) delivery, which leaves no foreign DNA in the final edited plant and produces edits indistinguishable from natural mutations [12,211].

Several countries, including the United States, Canada, Japan, Australia, Argentina, Brazil, and Israel, regulate such products by the characteristics of the final product. They consider genome-edited plants that contain no foreign DNA equivalent to conventionally bred crops and therefore not subject to GMO legislation [210,212]. In Latin America, nine countries have established similar frameworks, with Argentina as the pioneer, which makes the region the most accepting worldwide with respect to new technologies [210].

In contrast, the European Union has historically adopted a process-based regulatory approach [213,214]. Regarding GMOs, it maintains one of the strictest regulatory frameworks globally, grounded in the precautionary principle. This framework rests on Directive 2001/18/EC, which concerns the deliberate release of GMOs into the environment; Regulation (EC) No 1829/2003 [215], which is about genetically modified food and feed; and Regulation (EC) No 1830/2003 [216], which concerns traceability and labeling. Each GMO must undergo an extensive risk assessment by the European Food Safety Authority (EFSA) before approval and, as a result, very few crops are authorized for cultivation within the EU. Furthermore, mandatory labeling is required for products that contain GMOs above a threshold of 0.9%, while member states retain the right to prohibit the cultivation of approved GMOs [215,216,217]. Following a 2018 ruling by the Court of Justice of the European Union, plants that were produced through new genomic techniques (NGTs) were placed under GMO legislation and thus subjected to risk assessment, traceability and labeling [213,214]. However, in December 2025, the European Council reached a provisional agreement with the European Parliament on a new differentiated framework, which distinguishes NGT plants into two categories: Category 1, plants with minor modifications equivalent to conventional breeding, which are exempt from GMO legislation; and Category 2, plants with more complex modifications, which remain subject to the existing GMO regulatory framework [218]. As of the 17th of June 2026, the European Parliament and the Council formally adopted Regulation (EU) 2026/1388, establishing new rules that regulate plants based on their genetic profile rather than the technique that was used to produce them. The regulation will undergo a 2-year implementation period and is expected to foster innovation and support agrifood systems across the EU [219].

Additionally, countries such as China and Russia are developing similar legislative reforms that aim to distinguish genome-edited plants from GMOs [212]. It is also noteworthy that countries that have already approved transgenic plants are 22.6% more likely to approve genome-edited products as well [210].

Despite the rapid expansion of genome-editing research, relatively few edited crops have progressed from experimental development to sustained commercial use [220,221,222]. Among vegetable crops, the most prominent example is the high-GABA tomato commercialized in Japan. CRISPR/Cas9-mediated editing of SlGAD2 and SlGAD3 was shown to substantially increase GABA accumulation in tomato fruit [223], while the Sicilian Rouge High-GABA tomato subsequently entered the Japanese consumer market in 2021 [224]. In the United States, Pairwise introduced CRISPR-edited mustard greens with reduced pungency as Conscious Greens in 2023, although the product was later withdrawn as the company redirected its commercial activities [222]. These examples demonstrate that genome editing has progressed beyond proof-of-concept to market introduction, but also show that regulatory clearance and technical feasibility do not necessarily ensure sustained commercialization. Market demand, supply-chain development, protection of the variety or trait, access to investment, and the commercial strategy of the developer can all determine whether an approved genome-edited crop successfully reaches and remains on the market [221,222,225].

In summary, the global regulatory landscape for genetically modified and genome-edited plants remains without a unified and universally accepted framework; nonetheless, the international trend is gradually moving towards distinguishing such products from conventional GMOs [210]. Together with the emergence of the first commercially marketed genome-edited crops, these developments may facilitate broader adoption of genome editing in vegetable breeding. Nevertheless, regulatory approval alone does not ensure successful commercialization, which also depends on technical, economic and market-related factors [222].

## 6. Challenges and Constraints

Although the CRISPR/Cas system has made remarkable progress since 2020, it still faces several challenges in vegetable crops. One of the most substantial challenges that limits the broader adoption of genome editing in vegetable crops is the efficient delivery of CRISPR/Cas components into plant cells, which will subsequently lead to the regeneration of whole fertile plants. *Agrobacterium tumefaciens*-mediated transformation is by far the most widely used approach for a stable integration, with leaf discs, hypocotyl sections and cotyledon explants being used as the most common tissues. Additionally, transformation efficiency varies significantly between genotypes and species. Tomato and potato generally belong to the most amenable vegetables within the Solanaceae family, while many other vegetable crops exhibit varying degrees of recalcitrance [226,227] or are extremely difficult to transform. Even when transformation is achieved, the subsequent tissue culture and regeneration steps remain time consuming and complex [228]. Transgene-free editing strategies are increasingly important, both for regulatory reasons and for improved consumer acceptance. For that reason, two principal strategies have been employed. Initially, transient delivery of pre-assembled Cas9/sgRNA RNPs via protoplast transfection or biolistics enables editing without any DNA integration, minimizes off-target activity and shows reduced toxicity [5,229]. Additionally, it is possible to segregate the CRISPR/Cas transgene through selfing or crossing, for sexually propagated crops, as the editing construct and the edited locus reside at different chromosomal positions [8]. Ultimately, the creation of an efficient and universal transformation and regeneration system for vegetables poses challenges and is currently viewed as a limiting factor for wider CRISPR adoption [5,226]. Thus, new approaches, such as nanoparticle-mediated delivery and in planta transformation that bypass tissue culture, are promising alternatives. Finally, by using morphogenic regulators, such as the chimeric GRF4-GIF1 construct used in watermelon [204], transformation efficiency has improved in previously recalcitrant species.

As previously mentioned, plants with complex ploidy or large genomes may prove challenging to edit, as all alleles at a given locus must be disrupted simultaneously to achieve a homozygous genotype [5,199]. Off-target activity remains a concern and is directly affected by the dosage of Cas9 and sgRNA, the delivery method of the CRISPR construct to plant cells [12], and the production of regenerated plants. Therefore, to achieve a successful genetic transformation, editing efficiency must be considered, as it is influenced by the number of sgRNAs, content of GC, and expression levels of sgRNA and Cas9, as well as the secondary structure of the paired sgRNA and target sequence [227].

Beyond these technical limitations, intellectual property (IP) may represent an additional constraint for the commercial application of genome editing in vegetable breeding. The CRISPR/Cas patent landscape is complex, with rights covering core editing technologies and their improvements, and delivery and regeneration methods, as well as specific edited traits [230,231,232,233]. Consequently, vegetable breeders may face freedom-to-operate constraints and may need to obtain multiple licenses before commercially exploiting an edited trait [231,232]. Although licensing platforms can facilitate access to protected technologies, they do not necessarily ensure complete freedom to operate when multiple technology- and trait-related patents apply [231]. Thus, licensing requirements and associated legal complexity may increase development costs and partially offset the economic advantages of genome editing in commercial vegetable breeding [225,234]. Initiatives such as the International Licensing Platform—Vegetables (ILP) can facilitate access to patented traits, including genome-edited traits in some jurisdictions, although they do not necessarily ensure complete freedom to operate when additional technology-related patents apply [231].

## 7. Conclusions and Future Perspectives

CRISPR/Cas genome-editing research has been widely applied in the years 2020–2026 in various vegetable crops. Tomato and potato crops remain at the forefront and have drawn most of the research interest due to their importance, with the main edited trait for both crops being biotic stress resistance. Across trait categories, research that aims to confer biotic stress resistance, by targeting host susceptibility genes, is the dominant focus because of the enormous global losses that are imposed by pathogenic organisms on vegetable production. Some of the most significant advances of the past years are the *DMR6/S5H* gene family as a target for many crops to confer resistance to pathogens from different kingdoms, the *eIF4E* family for broad-spectrum viral resistance, and the *MLO* family for powdery mildew resistance across multiple species.

While traditional plant breeding approaches are time consuming and non-specific, plant breeding systems can be strengthened with the utilization of the CRISPR/Cas system and serve the current farmer and consumer needs. Despite the challenges the CRISPR/Cas system faces, it remains one of the most promising tools in vegetable crop breeding, and since it has such huge potential it is expected to overcome its remaining challenges and have a central role in meeting the demands of food security, nutritional quality and agricultural sustainability in a changing climate.

Despite remarkable progress, several technical challenges persist and remain laborious. Polyploidy requires the simultaneous disruption of all relevant homoeologous alleles and demands high efficiency in multiplex editing. Transformation recalcitrance in many vegetable species and genotypes continues to limit the speed at which validated targets can be transferred to commercial cultivars. Off-target activity remains a concern, although it has been reduced by high-fidelity Cas9 variants and RNP delivery. Therefore, the development of a truly efficient and universally applicable transformation and regeneration system for diverse vegetable crops is a critical priority.

Future applications of CRISPR/Cas in vegetable crop improvement include the adoption of multiple techniques. Prime and base editing can cause precise nucleotide substitutions and preserve gene function while they alter specific regulatory or catalytic residues. Multiplex editing can be utilized for the simultaneous stacking of multiple favorable traits within elite cultivar backgrounds in single transformation events; de novo domestication of wild relatives that carry valuable alleles, which confer stress tolerance or have nutritional value, and are absent from current breeding germplasm; and engineer chromosomal recombination to break the linkage drag that accompanies conventional introgression.

The current European regulatory system, which previously classified all genome-edited plants as GMOs, had long been a barrier to commercialization. However, the European Parliament and the Council reached a provisional political agreement in December 2025, which was formally adopted as Regulation (EU) 2026/1388 on 17 June 2026, marking a major shift toward a more permissive framework for certain genome-edited plants. This latest adoption of the new NGT legislation, alongside already permissive frameworks in other major regions, offers an increasingly favorable environment for the commercialization of CRISPR-edited vegetable crops in Europe.

## Figures and Tables

**Table 1:** Overview of the CRISPR/Cas toolbox: Cas variants, nuclease states, and the editing strategies they enable.

Cas System	Nuclease State	Target	Editing Strategy	DSB Required
Cas9	Active endonuclease	DNA	Knockout mutagenesis, multiplex editing	Yes
Cas12a, Cas12b, Cas12e	Active endonuclease	DNA	Knockout mutagenesis, multiplex editing	Yes
Cas14	Active endonuclease	Single-stranded DNA	Knockout mutagenesis, targeted cleavage	Yes
Cas13	Active endonuclease	RNA	RNA editing	Not applicable
Cas9 nickase and deaminase fusion	Catalytically impaired (nickase)	DNA	Base editing	No
Cas9 nickase and reverse transcriptase fusion, guided by pegRNA	Catalytically impaired (nickase)	DNA	Prime editing	No
Dead Cas9 (dCas9) + activator or repressor domain	Catalytically dead	DNA (regulatory region)	Fine-tuning of gene expression (CRISPRa/CRISPRi)	No
Active Cas9 or Cas12a and multiple gRNAs	Active endonuclease	DNA (multiple loci)	Multiplex editing, de novo domestication	Yes
Cas9 or Cas12a fused to Pong transposase (ORF1–ORF2)	Type II or V, transposase-linked	Active endonuclease (Cas) and transposase (cut-and-paste insertion)	DNA (large insertions)	CRISPR-transposase insertion
Cas13a (e.g., LwaCas13a)	Type VI	Active endonuclease with collateral (trans-)cleavage activity	RNA	Nucleic acid diagnostics (SHERLOCK)

## Data Availability

No new data were created or analyzed in this study. Data sharing is not applicable to this article.
